# Health economic evaluation of blended collaborative care for older multimorbid heart failure patients: study protocol

**DOI:** 10.1186/s12962-024-00535-2

**Published:** 2024-04-13

**Authors:** Lisa Derendorf, Stephanie Stock, Dusan Simic, Arim Shukri, Christine Zelenak, Jonas Nagel, Tim Friede, Birgit Herbeck Belnap, Christoph Herrmann-Lingen, Susanne S. Pedersen, Jan Sørensen, Dirk Müller and on behalf of the ESCAPE consortium

**Affiliations:** 1 Faculty of Medicine and University Hospital of Cologne, Institute of Health Economics and Clinical Epidemiology, University of Cologne, Cologne, Germany; 2 Department of Psychosomatic Medicine and Psychotherapy, University of Göttingen Medical Centre, Göttingen, Germany; 3 German Centre for Cardiovascular Research (DZHK), Partner Site Göttingen, Göttingen, Germany; 4 Department of Medical Statistics, University Medical Centre Göttingen, Göttingen, Germany; 5 Center for Behavioral Health, Media, and Technology, Division of General Internal Medicine, University of Pittsburgh School of Medicine, Pittsburgh, PA USA; 6 Department of Psychology, University of Southern Denmark, Odense, Denmark; 7 Department of Cardiology, Odense University Hospital, Odense, Denmark; 8 Healthcare Outcomes Research Centre, Dublin, Ireland

**Keywords:** Health economic evaluation, Cost-effectiveness, Cost-utility, Heart failure, Multimorbidity, Collaborative care, Time-driven activity-based costing, Depression, Emotional distress, Elderly

## Abstract

**Background:**

Integrated care, in particular the ‘Blended Collaborative Care (BCC)’ strategy, may have the potential to improve health-related quality of life (HRQoL) in multimorbid patients with heart failure (HF) and psychosocial burden at no or low additional cost. The ESCAPE trial is a randomised controlled trial for the evaluation of a BCC approach in five European countries. For the economic evaluation of alongside this trial, the four main objectives were: (i) to document the costs of delivering the intervention, (ii) to assess the running costs across study sites, (iii) to evaluate short-term cost-effectiveness and cost-utility compared to providers’ usual care, and (iv) to examine the budgetary implications.

**Methods:**

The trial-based economic analyses will include cross-country cost-effectiveness and cost-utility assessments from a payer perspective. The cost-utility analysis will calculate quality-adjusted life years (QALYs) using the EQ-5D-5L and national value sets. Cost-effectiveness will include the cost per hospital admission avoided and the cost per depression-free days (DFD). Resource use will be measured from different sources, including electronic medical health records, standardised questionnaires, patient receipts and a care manager survey. Uncertainty will be addressed using bootstrapping.

**Discussion:**

The various methods and approaches used for data acquisition should provide insights into the potential benefits and cost-effectiveness of a BCC intervention. Providing the economic evaluation of ESCAPE will contribute to a country-based structural and organisational planning of BCC (e.g., the number of patients that may benefit, how many care managers are needed). Improved care is expected to enhance health-related quality of life at little or no extra cost.

**Trial registration:**

The study follows CHEERS2022 and is registered at the German Clinical Trials Register (DRKS00025120).

**Supplementary Information:**

The online version contains supplementary material available at 10.1186/s12962-024-00535-2.

## Introduction

Heart failure (HF) is a major global health challenge, affecting more than 64 million people worldwide [1]. It is a leading cause of mortality, morbidity, hospitalisation and healthcare costs, accounting for 1–3% of the total healthcare expenditure in European countries [2]. HF significantly impairs health-related quality of life (HRQoL) and is often associated with co-morbid other somatic and/or mental disorders [1, 3]. Integrated care has shown to be an effective way of dealing with the complexity of multimorbidity.

Various forms of integrated care, such as collaborative care (CC) or blended collaborative care (BCC), have shown to enhances primary care for individuals with anxiety disorders and depressive symptoms [4–6]. In addition, several sources suggest that integrated care approaches could lead to a reduction in care expenses thus offsetting (at least in part) the intervention costs [5, 7–12]. This reduction is assumed to result from the decline in formal caregiver visits (e.g. through improved coordination), which in turn is associated with fewer medical complications or hospitalisations [12, 13].

CC involves active follow-up by non-physician care managers who supports patients (e.g., by coordinating contacts, educating them about their illness, or proactively monitoring their responses to therapy). In contrast to CC which targets somatic or mental disorders, BCC interventions provide care for both conditions simultaneously [6, 14]. BCC has shown to improve both mental and physical health in patients affected by chronic somatic diseases with comorbid psychological distress such as anxiety disorders or depressive symptoms [6]. However, existing research in this area is limited and BCC interventions have not been specifically evaluated yet in European multimorbid patient populations [4].

The project ‘Evaluation of a patient-centred biopSychosocial blended collaborative CAre Pathway for the treatment of multimorbid Elderly patients (ESCAPE)’ (Horizon 2020 No. 945377) aims to develop and evaluate an integrated care pathway tailored to elderly patients experiencing somatic-mental multimorbidity, with a specific focus on HF patients [15]. The ESCAPE project includes a randomized-controlled trial (RCT) and a cohort study; both are taking place across eleven clinical centres in five European countries, including Denmark (Odense, Roskilde and Slagelse), Germany (Göttingen, Cologne, Leipzig, and Hamburg), Hungary (Budapest), Italy (Bologna), and Lithuania (Kaunas).

## Study objectives

The health economic part of ESCAPE was designed to investigate the economic impact of the BCC intervention and transfer these findings across the different jurisdictions represented by the participating countries. Furthermore, the results will support decision-making regarding the implementation of the BCC intervention. Specifically, the analysis aims to achieve the following:

1. To document the provision costs of the intervention,
2. To assess the running costs of the intervention at the different trial sites,
3. To evaluate the short-term cost-effectiveness and cost-utility of the ESCAPE BCC plus usual care intervention compared with usual care alone (based on the RCT data),
4. To examine the budgetary implications of implementing the intervention in different health care systems practice.

## Methods and analysis

### Study design, study setting and selection of participants

Following the SPIRIT reporting guidelines, the study structure is described as a comprehensive cohort study including an embedded RCT. A more detailed description of the ESCAPE trial can be found in Zelenak et al. [15]. The RCT’s primary focus is to determine whether the addition of a 9-month optimised and targeted BCC intervention in elderly multimorbid patients to UC improves HRQoL compared to UC alone. For each treatment group, 150 patients aged 65 years or older with all types of confirmed HF, ≥ 2 chronic somatic comorbidities, and psychological distress or mental disorder were expected to be enrolled in the study. Among the five participating countries, the intended target enrolment target was assumed to range between 33 and 124 patients. In the intervention group, trained care managers supervised by multidisciplinary specialist teams provide pro-active support to patients and their informal carers to effectively manage their multiple health problems.

The trial is registered at the German Clinical Trials Register (DRKS00025120). The recruitment started at the coordinating site in Göttingen, Germany, in April 2022, with subsequent sites joining in the subsequent twelve months. The first patient was randomized in July 2022, and recruitment efforts are ongoing.

### Health economic analyses

Health economic evaluations are systematic assessments of the costs and effects of healthcare interventions or strategies. Their primary purpose is to inform decision-making by providing quantitative evidence on the comparative efficiency and value of different health care options [16, 17]. In the ESCAPE study, the trial-based economic analyses will include both a cost-effectiveness analysis (CEA) and a cost-utility analysis (CUA) with subgroup analyses for (i) depressive symptoms vs. no depressive symptoms and (ii) cardiovascular vs. no cardiovascular symptoms [16]. Evaluations will be made from a payer perspective.

### Cost-utility analysis

Where sufficient data are available, the calculation of QALYs for the CUA is done on a country-by-country basis using the EQ-5D-5L. The chosen index will be informed by national value sets specific to each country. As there is no value set for Lithuania, the closest available value set, i.e., Polish values, will be used due to its geographical proximity [18].

### Cost-effectiveness analysis

As the expected benefit of the BCC intervention may not be reflected in a corresponding gain in QALYs, two additional CEAs will be performed: (i) for all patients, the cost per hospital admission avoided; (ii) for individuals with a given level of depressive symptoms, the cost per depression-free day (DFD), with DFDs as the number of days per annum a patient is depression-free. The different incremental cost-effectiveness ratios (ICERs) will be calculated as follows:

The cost per hospital admission avoided is calculated by comparing the healthcare utilisation of the BCC intervention with that of UC alone and across different subgroups with cardiovascular symptoms identified from the Kansas City Cardiomyopathy Questionnaire (KCCQ-12). The calculation of the DFDs will be based on data acquired from the HADS. Calculating DFDs, the translation of the HADS scores into DFDs will be based on the conversion method outlined by Lave et al. [19]. Details of the conversion method are outlined in the section ‘Subgroup analyses’.

**Fig. 1:**
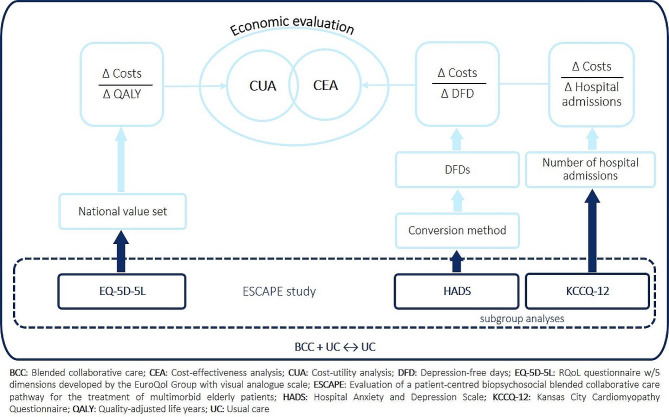
Planned economic evaluation in the ESCAPE study

Figure 1 provides an overview of the intended structure of the economic evaluation, and each aspect is explained in detail below.

### Data collection

#### Outcome measures

Clinical effects will be measured using the EQ-5D-5L, HADS, and KCCQ-12 instruments at three specific time points: during the baseline assessment and randomisation phase (BL), at the ‘end of treatment’ in the RCT after 9 months (FU1), and finally, at the ‘end of study’ evaluation, which will take place 18–33 months after enrolment (FU2).

The **EQ-5D-5L** index will be used as a tool to gauge QALYs [20, 21]. The EQ-5D-5L, designated as the primary outcome of the main study, includes dimensions such as mobility, self-care, usual activities, pain/discomfort, and anxiety/depression. It has been used in a wide range of studies across Europe and in different populations including multimorbid and older people [22, 23]. This index has been linguistically validated and exhibits satisfactory sensitivity to change.

The **KCCQ-12** is a concise 12-item patient-reported instrument designed to assess the quality of life and functional status of individuals with HF. It quantifies various domains, such as physical limitations, symptoms, self-care, and social limitations, providing a comprehensive picture of a patient’s cardiac health-related well-being [24].

The **HADS** will be used to assess a patients’ psychological distress. This scale, which has also been used in medical studies with patients with heart disease, produces separate scores for severity of anxiety and depression symptoms [25]. Overall psychological distress is measured by the total score, ranging from 0 to 42, with a score exceeding 12 assumed to detect at least mild levels of prognostically relevant distress.

### Resource use and costs

Data on resource use and costs associated with the BCC intervention will be obtained from multiple sources and standardised approaches, as the feasibility of accessing medical records and the costs vary from country to country. The imergo® e-health Integrated Care Platform (ICP) will be used to collect study-related medical information and to further enhance the data collection process for patients assigned to the intervention group throughout the study. Patient-related and provider-related utilisation will be measured by applying a time-driven activity-based costing (TDABC) approach [26]. The TDABC will streamline the care process by focusing on the time required to execute activities along the care pathway. Corresponding costs will be assigned to specific activities by multiplying the time invested in an activity by the corresponding capacity cost rate of the resources involved [27–30]. Table 1 provides an overview of possible methods for collecting costs and resources.

**Table 1:** Potential methods for collecting costs and resources

Sites	Patient-related		Provider- and study-related	
	Health insurance data (utilisation and costs)	Medical consumption (only utilisation)	imergo® e-health ICP (study-related medical records and assessments)	Care manager survey (provider-related resources)
Germany	Patient receipts	-	x	x
Denmark	Possible	iMCQ	x	x
Italy	tbd	iMCQ	x	x
Lithuania	tbd	iMCQ	x	x
Hungary	tbd	iMCQ	x	x

### Patient-related utilisation: medical consumption questionnaire (iMCQ)

Patient-related resource utilisation will be assessed using the iMCQ, medical records and study data retrieved from the external clinical information system secuTrial and the imergo® e-health ICP. The iMCQ will cover all healthcare services provided to individual patients which are not part of the intervention. In order to reduce the participants’ burden of study-specific processes, questions of limited relevance were excluded in accordance with the prescribed questionnaire guidelines [31]. Similarly, the medication chart has been adapted as the task of documenting medication details will be entrusted to the care managers. The iMCQ will be administered at the start of the RCT, at the 9-month point (i.e., end of treatment), and at the ≥ 18-month milestone (i.e., end of study). The retrospective iMCQ will record the patient’s medical consumption over the last 3 months. As it is not feasible to administer the iMCQ quarterly, patient-related utilisation will be determined based on available evidence and agreed upon by clinical experts. Resource utilisation will be extrapolated to the 9-month intervention or 18 to 33-month follow-up period. The iMCQ will not be implemented in Germany, as patient receipts cover costs and medical consumption.

### Provider-related utilisation: care manager survey

The care manager survey is intended for documenting the resource use following the services provided by the care managers. This requires each care manager to record the time spent on the various activities that comprise the BCC intervention. For example, the survey covers time spent on patient contact, organisation of treatment schedules and follow-up, collaboration with general practitioners, specialists, and informal carers, as well as other organisational and administrative tasks. The survey will take place over a period of four weeks and spread over different time points at all participating sites. This approach aims to neutralise potential seasonal effects and variation within patient cohorts, as well as to incorporate provider learning and scale effects [32]. In addition to the primary care manager survey, a short supplementary questionnaire will be administered to the local trainers of care managers in each country. This supplementary questionnaire is designed to capture the time spent on tasks related to training, educational initiatives, and the regular case review meetings conducted for supervision purposes. The questionnaires have been developed and subjected to pre-testing (see Additional file 1). In addition, imergo® e-health ICP data on the number and duration of care management contacts will be used.

### Medical costs and costs of providing the intervention

To facilitate a later implementation of BCC in practice, costs will be categorised into (i) those associated with the resource use and treatment of patients in the ECAPE intervention and (ii) those associated with the provision and maintenance of the intervention. Costs associated with patient resource use and treatment will be collected through iMCQ and/or health insurance data or flat rates for services. In Germany where claims data on patients’ medical costs will be available, these sources will be used to further corroborate the information obtained from the questionnaires, such as the iMCQ. This dual approach ensures data validation and should increase the accuracy and reliability of the economic evaluation. In Germany, patient receipts (based on Sect. 305 SGB V) include information on the services patients have received and the corresponding costs. German health insurance funds are obliged to provide these receipts to insured individuals, upon request. The participant signs an additional informed consent form that legally authorises the study staff to request data from health insurance companies. The subsequent data request and delivery process between the study team and the health insurance companies for all German sites will be centrally coordinated by the study centre in Göttingen in accordance with national regulations and data protection laws. Statutory health insurance (SHI) access is likely to be feasible mainly in Germany. Where cross-country comparisons are possible, results are converted into a common currency using purchasing power parity adjustments in order to account for differences in the cost of goods and services across countries [33].

The costs related to the provision of the intervention will reflect the resource use associated with the BCC intervention, i.e., costs related to the time spent by care managers, costs due to training and those of supervision. In addition, the costs of information and education materials for patients and their informal carers and the costs associated with the technological support (including the imergo® e-health ICP, network websites and interactive, multilingual patient websites) will also be considered.

**Fig. 2:**
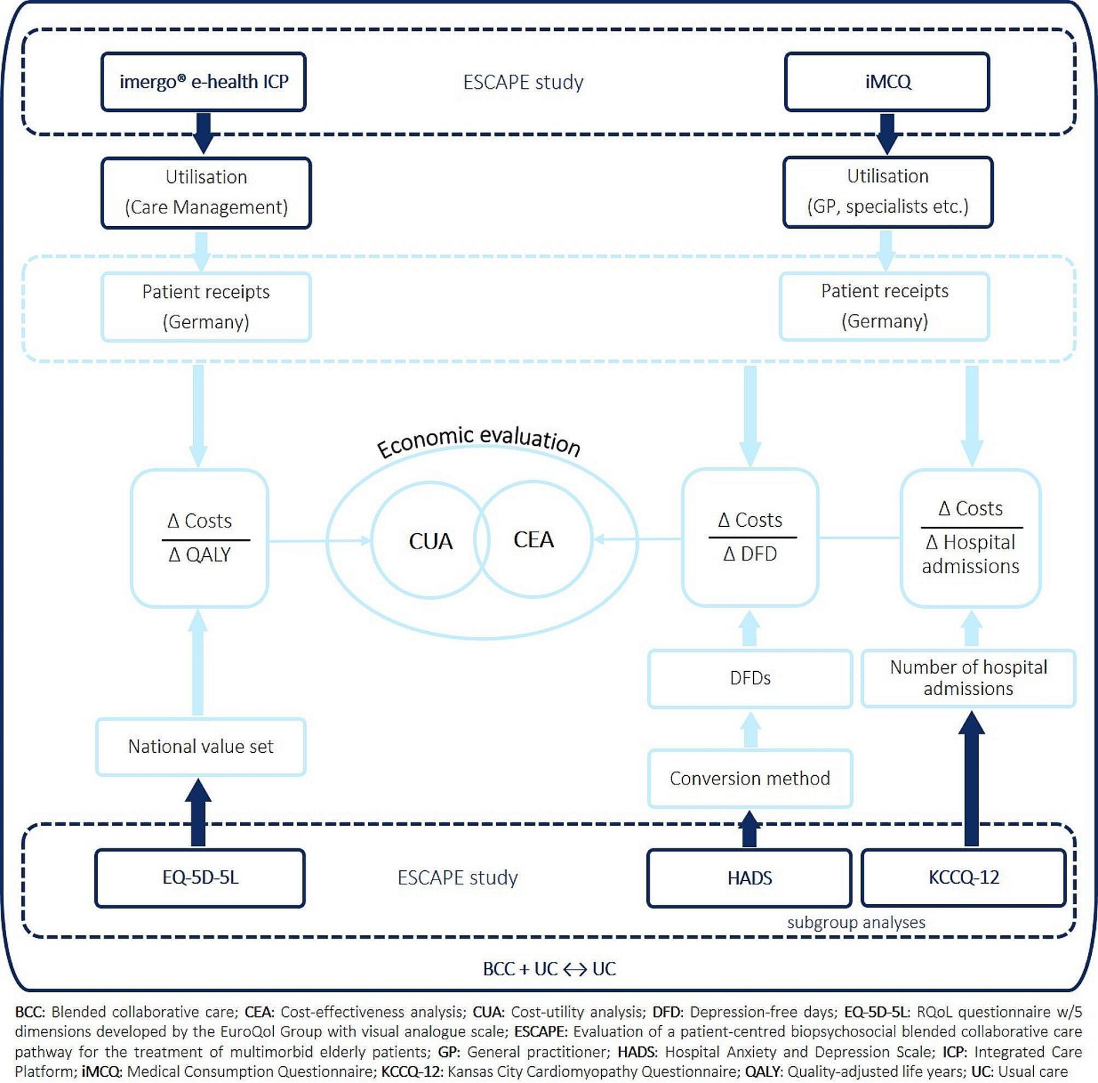
Planned economic evaluation in the ESCAPE study including data collection measures

Figure 2 provides a detailed overview of the intended structure of the economic evaluation, including the outcome measures, and the identification of resource use and costs.

### Uncertainty and validity

Addressing uncertainty in economic evaluations is crucial for enhancing the credibility of findings. Sensitivity analyses provide transparency about the robustness of the results, helps decision-makers to understand the potential variability in outcomes, and enables informed decisions by considering the range of possible scenarios and their associated risks and benefits. Bootstrap nonparametric resampling will be used to account for uncertainty in the analyses [34]. It allows drawing samples with replacement from the original data for calculating the parameter of interest repeatedly. This generates a vector of bootstrap replicates, serving as an empirical estimate of the statistic’s sampling distribution. Repeating this process numerous times (at least 1000 times for confidence intervals) generates the empirical distribution of cost-effectiveness [34]. The results will be presented using cost-effectiveness planes and cost-effectiveness acceptability curves (CEAC). The cost-effectiveness plane serves as a visual representation, highlighting random values of both incremental cost and effects. The CEAC provides the probability of cost-effectiveness of the intervention across a range of willingness-to-pay thresholds [35]. CEACs allow decision-makers to calibrate their judgements across different financial thresholds.

To strengthen the internal and external validity of this study, the Consolidated Health Economic Evaluation Reporting Standards 2022 (CHEERS2022) statement will be followed [36].

### Subgroup analyses

All outcome measures, i.e., EQ-5D-5L, HADS and KCCQ-12, will be assessed in all patients at three time points (baseline, end of treatment and end of study). Additional subgroup analyses for patients with depressive and cardiovascular symptoms will be performed (see Table 2). For patients with cardiovascular symptoms (e.g., shortness of breath, fatigue), the KCCQ-12 will identify subgroups related to symptom severity. Subgroup analyses will be performed based on scores less than 75 (very poor to good) and 75 to 100 (good to excellent) [37, 38].

DFDs will be calculated for patients with a score of 8 or higher on the HADS depression subscale to identify patients with depressive symptoms [39]. For the HADS total scale, the cut-off will be 15 and above [40]. HADS scores will be converted to DFD using linear interpolation. This method has been used in previous studies, for example with the Hopkins Symptom Checklist (HSCL) [7, 19, 41]: Over the course of a follow-up period, patients were assigned an appropriate DFD value for each day. To estimate the DFD value, scores of the HSCL that are equal to or less than 0.50 were attributed one DFD, scores of 1.7 or greater were assigned zero DFDs, and scores between 0.50 and 1.7 were assigned DFD values ranging from 0 to 1 by linear interpolation. For example, an HSCL-20 score of 1.1 corresponds to 0.5 DFD [41]. It is noteworthy that these thresholds values have been validated by previous research (i.e. correlation with the Hamilton Rating Scale-Depression (HRS-D) scores or the Beck Depression Inventory (BDI)) [42, 43]. However, until now there has been no formula for converting measures of severity of disease-specific symptoms (e.g., a patient’s psychological distress) using the HADS. As part of this analysis, a formula, and thresholds for converting the HADS to DFDs will be developed and defined. In addition, DFDs can be used to validate QALYs. This approach is based on the rationale that depression is equivalent to a 0.2–0.4 reduction in QALY weights, so that 1 year of depression is effectively equivalent to an equivalent reduction in QALYs [43–45].

**Table 2:** Overview of outcome measures and subgroup analyses

Type of analysis	Questionnaire	Endpoints		Characteristics of subgroup
		All Patients	Subgroup	
**Cost-effectiveness analysis** **(CEA)**	KCCQ-12	Hospital admissions avoided	Hospital admissions avoided	Cardiovascular symptoms as measured by the KCCQ-12 with a cut-off score ≤ 74.
	HADS	-	Depression-free days	Depressive symptoms as measured by the depression subscale of the HADS at a cut-off of ≥ 8 and for the HADS total score at ≥ 15.
**Cost-utility** **analysis** **(CUA)**	EQ-5D-5L	QALYs	-	-

## Discussion

This study protocol presents a comprehensive design for the health economic evaluation of a BCC intervention for elderly, multimorbid HF patients. By exploring the economic dimensions, this evaluation aims to provide insights into the practical feasibility, cost-effectiveness, and wider implications of implementing BCC intervention to elderly multimorbid HF patients across Europe.

One strength of this study is that the economic analysis will be based on various methods and approaches. For example, the integration of a TDABC approach enables an accurate identification of cost drivers by directing attention towards time. This direct alignment between cost and activity duration not only facilitates a detailed reflection of the care process but also allows for a streamlined implementation of BCC intervention in routine care contexts [27, 29]. Moreover, the TDABC has shown to be adaptable to different international health care settings [26, 29].

The economic assessment of the BCC intervention faces various methodological challenges. However, provided sufficient data, the chosen design may serve as a solid basis for the evaluation. Standardised questionnaires, including three outcome assessments, are a viable basis for assessing the quality of life and resource use of patients with HF receiving BCC.

Although our economic approach for evaluating BCC will be based on a variety of methodologies, there are two major limitations. First, as it is common for trial sample sizes to be based on the primary clinical outcome, the analysis lacks a pre-planned sample size calculation specifically for the economic aspects [33]. This could impact the statistical power of the economic evaluation (e.g., due to a higher extent of incompleteness for cost data than for clinical data). Second, the uncertainty of obtaining consistent cost data for all five European countries, and the wide variation in target recruitment numbers between countries, may weaken the robustness of the analysis. As a result, and facing the inherent uncertainties of such complex projects, partial changes to the analysis plan may be justified and unavoidable.

In conclusion, the comprehensive cross-country approach of the analysis, combined with a sophisticated manifoldness in the methods used for determining costs and outcomes, should provide a substantial starting point for a high-quality economic study on BCC intervention for older multimorbid patients. Providing the economic evaluation of ESCAPE will contribute to a country-based structural and organisational planning of BCC (e.g., the number of patients that may benefit, how many care managers are needed). The anticipated release of primary results is in 2026.

### Electronic supplementary material

Below is the link to the electronic supplementary material.

Supplementary Material 1

## Abbreviations

(B)CC: (Blended) collaborative care
BDI: Beck Depression Inventory
BL: Baseline
CEA: Cost-effectiveness analysis
CEAC: Cost-effectiveness acceptability curve
CHEERS: Consolidated Health Economic Evaluation Reporting Standards 2022 (CHEERS2022) Statement
CUA: Cost-utility analysis
DFD: Depression-free days
EQ-5D-5L: RQoL questionnaire w/5 dimensions developed by the EuroQol Group
ESCAPE: Evaluation of a patient-centred biopsychosocial blended collaborative care pathway for the treatment of multimorbid elderly patients
EU: European Union
FU: Follow-up
GP: General practitioner
HADS: Hospital Anxiety and Depression Scale
HRS-D: Hamilton Rating Scale-Depression
HF: Heart failure
HRQoL: Health-related quality of life
HSCL: Hopkins Symptom Checklist
ICP: Integrated care platform
ICER: Incremental cost-effectiveness ratio
iMCQ: Medical Consumption Questionnaire
ISPOR: International Society for Pharmacoeconomics and Outcomes Research
KCCQ-12: Kansas City Cardiomyopathy Questionnaire
MAM: Meta-algorithm for multimorbidity
PIC: Patient-informed consent
QoL: Quality of life
QALY: Quality-adjusted life years
RCT: Randomized controlled trial
RCT-CEA: Randomized Clinical Trials-Cost-Effectiveness Analysis
SHI: Statutory Health Insurance
TDABC: Time-driven activity-based costing
UC: Usual care
