# Supplementary material for: Health economic evaluation of blended collaborative care for older multimorbid heart failure patients: study protocol
Source: Cost Eff Resour Alloc. 2024 Apr 13;22:29. doi: 10.1186/s12962-024-00535-2 (PMC11015692; doi:10.1186/s12962-024-00535-2)
Supplement: Supplementary file 1 — Supplementary Material 1 [file 12962_2024_535_MOESM1_ESM.pdf]

ESCAPE BCC – Time-related resource consumption

Part 1: Care Manager

The aim of this survey is to identify **time-related resource consumption** for the ESCAPE Blended Collaborative Care (BCC) intervention that is not documented in the electronic registry in your clinical routine. The results will be included in the economic analysis.

How to do it:

After 3-4 months of experience with the intervention, choose a 4-week window that is not interrupted by holidays and vacations. Please complete the time recording for all tasks as outlined below for your working days in the ESCAPE BCC intervention during this period.

It is important to **briefly record the time spent on the various tasks and activities** of your working day on the attached timesheet. You can print out this Word timesheet and keep it next to your workstation.

Choose what works best for you. Whichever solution you choose, please remember to use **a new Word timesheet for each new working day and patient-ID**.

Please note that some days may be very short, e.g. just trying to reach a GP, and other days you may work for several hours. We are not interested in how much time you spent with a single patient during the whole intervention, but how much time you spent per day on the different tasks. Note that not all activities will take place every day. On the other hand, some tasks may need to be done several times during a working day, e.g. phone calls to GPs. If an activity occurs more than once a day, you can add the times together or just enter several times. Leave the fields blank for activities that do not occur.

Exact times are nice to have and should be aimed for. We understand that this is not always possible, as sometimes there are unexpected interruptions in the daily work routine, occur more than once a day, or are difficult to track. In this case, you can add the times together or simply **enter several times** (e.g. 2 min. / 5 min. / 8 min. / 1 min.). We will add them up for you.

Do **not include your time spent on this survey or purely study-related activities** (e.g., SAE recording, correcting input from secutrial, RCT baseline data entry into secutrial).

Definitions and examples of what is included in the different survey categories:

Preparation time	Contact	Follow-up
**Definition**		
Time spent preparing any kind of contact.	The actual length of time spent communicating with one or more people.	Time spent following up on a previous contact or resulting Tasks.
**Examples**		
– Time spent **contacting** the GP or GP office **several times by telephone.**<br>– Times spent **searching for information** on the internet.<br>– Times spent finding community resources.<br>– Time spent **maintaining** MAM.<br>– Time spent **writing reports** or updates.<br>– Time spent **preparing a presentation** of your patient's case for the case review.	– Time spent **communicating** with the GP or GP office.<br>– Time spent **talking** to informal carers.<br>– Time spent on the **phone** with community specialists or their offices.<br>– Time spent **emailing** with community resources.<br>– Time spent presenting and **discussing** your patient's case.	– Time spent **documenting in the registry** following patient contact.<br>– Time spent **searching for community resources suggested in the case review** meeting.

At the end of the survey period, we will contact you to clarify the transfer of the collected timesheets. Please do not hesitate to contact us if you have any questions.

ESCAPE BCC – Timesheet

**Date:**	**Patient-ID:**

Recruitment site: ☐ Göttingen ☐ Cologne ☐ Hamburg ☐ Leipzig ☐ Odense ☐ Kaunas ☐ Roskilde ☐ Slagelse ☐ Budapest ☐ Dublin ☐ Bologna

**Patient contact**	Duration in minutes
**Preparation:** Time spent preparing for contact with the patient.	
**Contact:** Time spent communicating with the patient.	
**Follow-up:** Time spent following up the contact with the patient.	
**Collaborating with treating care (GP or GP office)**	
**Preparation:** Time spent preparing to work with the treating care.	
**Contact:** Time spent for communicating with the treating care.	
**Follow-up:** Time spent following up the contact with the treating care.	
**Collaborating with treating care specialists (e.g., Cardiologist)**	
**Preparation:** Time spent preparing to work with the treating care specialists.	
**Contact:** Time spent for communicating with the treating care specialists.	
**Follow-up:** Time spent following up the contact with the treating care specialists.	
**Involving informal care givers**	
**Preparation:** Time spent preparing to involve informal care givers.	
**Contact:** Time spent for communicating with the informal care givers.	
**Follow-up:** Time spent following up the contact with the informal care givers.	
**Connecting patients to external resources**	
**Time spent** researching on external resources (e.g., searching for programs or self-help groups, including contacting and documenting).	
**Reminders**	
**Time spent** sending personalised text messages and reminders between calls (including the contact and documenting the contact).	
**Special events** (**no study-related activities**)	
**Time spent** on caring for and coordinating patients with special events (e.g., suicide protocol).	

Case review	Duration in minutes
**Preparation:** Total amount of time spent preparing your cases for the case review (e.g., listing of patients that need to be discussed, preparing the case presentation).	
**Follow-up:** Total amount of time spent following up the case review contact (e.g., documenting the results of case review discussions).	
**Reporting**	
**Time spent** working on any type of report (e.g. interim and final reports).	
**Others**	
Are there any activities that are not documented in this timesheet or in the electronic registry but that require time-related resources? If so, please provide a brief description of the activity and the time involved.	

ESCAPE BCC – Time-related resource consumption

Part 2: Trainer

The aim of this survey is to identify **time-related resource consumption** for the ESCAPE Blended Collaborative Care (BCC) intervention that is not documented in the electronic registry in your clinical routine. The results will be included in the economic analysis.

How to do it:

Please complete the time recording for the tasks as outlined below the ESCAPE BCC intervention. It is important to **briefly record the time spent on the various tasks and** on the attached timesheet. If possible, please also list all past events.

Exact times are nice to have and should be aimed for. We understand that this is not always possible, as sometimes there are unexpected interruptions in the daily work routine, occur more than once a day, or are difficult to track. In this case, you can add the times together or simply **enter several times** (e.g. 2 min. / 5 min. / 8 min. / 1 min.). We will add them up for you.

Do **not include your time spent on this survey or purely study-related activities**.

At the end of the intervention, we will contact you to clarify the transfer of the collected timesheets.

Please do not hesitate to contact us if you have any questions.

ESCAPE BCC – Timesheet Part 2

**Recruitment site:**	☐ Göttingen ☐ Cologne	☐ Hamburg ☐ Leipzig	☐ Odense ☐ Kaunas	☐ Roskilde ☐ Slagelse	☐ Budapest ☐ Dublin	☐ Bologna				
**Date:**										
**Case review**										
**Contact:** Time spent presenting and discussing patient cases at the case review.										
**Training and education**										
**Time spent** on care manager training and education (e.g., online training, supervision, etc.)										
**Preparation and follow-up time of care managers** for training and education (if applicable)										
**Others** (Please provide a brief description of any activities that might require time-related resources and are not documented here.)										
